# Ileal Bile Acid Transporter Inhibition for the Treatment of Chronic Constipation, Cholestatic Pruritus, and NASH

**DOI:** 10.3389/fphar.2018.00931

**Published:** 2018-08-21

**Authors:** Samer Al-Dury, Hanns-Ulrich Marschall

**Affiliations:** Wallenberg Laboratory, Department of Molecular and Clinical Medicine, Sahlgrenska Academy, Institute of Medicine, University of Gothenburg, Gothenburg, Sweden

**Keywords:** ASBT, bile acids, cholestasis, C4, FGF19, IBAT, pruritus

## Abstract

Bile acids are synthesized from cholesterol in the liver, excreted with bile into the duodenum, almost completely taken up again in the distal ileum and finally returned to the liver with portal blood in a process termed enterohepatic circulation. Bile acid synthesis, excretion, and reuptake are tightly regulated. The apical sodium-dependent bile acid transporter [ASBT; also known as ileal bile acid transporter (IBAT) and SLC10A2] is pivotal for the almost complete reabsorption of conjugated bile acids in the ileum. Dysfunctional IBAT may be the cause of bile acid diarrhea. Pharmacological IBAT inhibition results in an increased bile acid load in the colon and subsequently a lower bile acid pool, which is associated with improved liver histology in animal models of cholestatic liver disease and non-alcoholic steatohepatitis (NASH). In humans, IBAT inhibitors have been tested in clinical trials with widely different indications: in patients with idiopathic chronic constipation, an increased number of bowel movements was observed. In adult and pediatric cholestatic liver diseases with pruritus, various IBAT inhibitors showed potential to improve itching. Adverse events of IBAT inhibitors, based on their mode of action, are abdominal pain and diarrhea which might patients to withdraw from study medications. So far, no data are available of a study of IBAT inhibitors in patients with NASH. In this review we summarize the preclinical and most recent clinical studies with various IBAT inhibitors and discuss the difficulties that should be addressed in future studies.

## Enterohepatic Circulation and Feedback Control of Synthesis of Bile Acids

**FIGURE 1:**
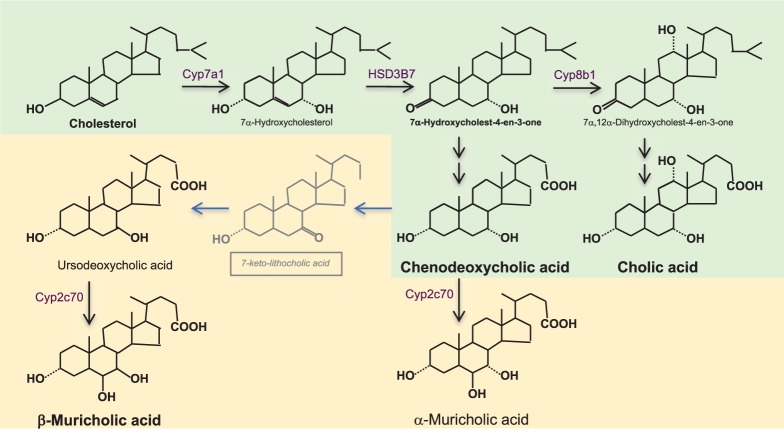
Bile acid synthesis in the liver. Bile acids (BAs) synthesis from cholesterol synthesis requires almost 20 enzymatic reactions in different subcellular compartments of the hepatocyte. The rate limiting enzyme is cholesterol 7α-hydroxylase (CYP7A1). Its activity is reflected by serum 7α-hydroxy-4-cholesten-3-one (C4) which is formed by the action of 3β-hydroxy-Δ^5^-C_27_-steroiddehydrogenase/isomerase (HSD3B7). In humans (shown in green), the primary BAs are chenodeoxycholic acid (CDCA) and cholic acid (CA); the ratio between them is determined by 12α-hydroxylase (CYP8B1), which is required for the formation of CA. In rodents (shown in yellow), there are additional primary BAs; ursodeoxycholic acid (UDCA) and α-and β-muricholic acids (α/β MCA). α/βMCAs are generated by 7β-hydroxylation of CDCA and UDCA by cytochrome CYP2C70 while the mechanism of epimerization of CDCA toward UDCA still is unknown. BAs are conjugated with glycine- or taurine (in humans) and with taurine (in rodents) before excretion into bile. Murine BA profiles are much more hydrophilic than human BA profiles and have substantially different activation proprieties of the nuclear BA receptor farnesoid X receptor (FXR, see **Figure 2**). While CDCA is the strongest natural activator of FXR, taurine-conjugated α/βMCAs are natural antagonists of FXR, which one needs to keep in mind when translating BA-related metabolic data from rodents to humans.

**FIGURE 2:**
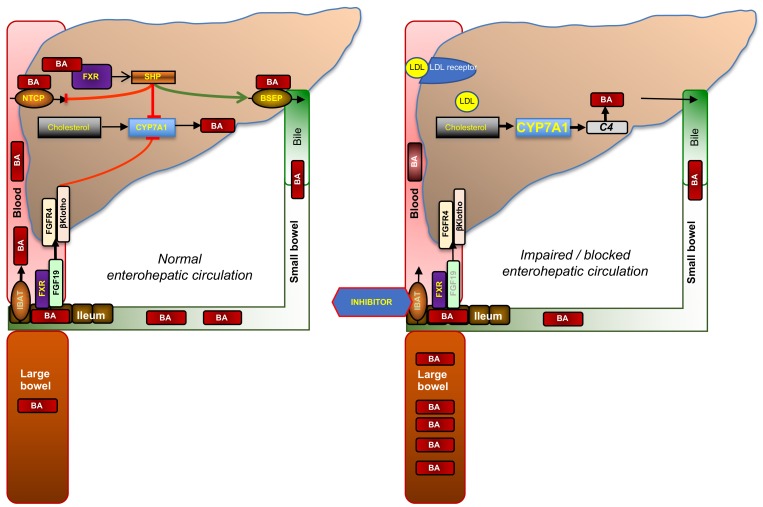
Enterohepatic circulation of bile acids and mode of action of ileal bile acid transporter (IBAT) inhibitors. Glycine- or taurine- conjugated BAs are excreted via the bile salt export pump (BSEP) into bile in which they reach the duodenum. About 95% of conjugated BAs are reabsorbed in the terminal ileum via the apical sodium-dependent BA transporter (ASBT, *SLC10A2*; also known as IBAT, ileal BA transporter) and recirculated to the liver via the portal vein from which they are reabsorbed via the sodium-dependent taurocholate co-transporting peptide (NTCP, *SLC10A1*). This recycling process is called enterohepatic circulation. The rate-limiting enzyme of BA synthesis from cholesterol, CYP7A1, is controlled by two different negative feed-back pathways that are both regulated by binding of BAs to its nuclear receptor, FXR: (1) within the liver, when BA exceed physiological levels as in cholestasis, via small heterodimer partner (SHP) that signals to reduced expression of CYP7A1 and NTCP, and increased expression of BSEP; and (2) from the ileum via increased formation of fibroblast growth factor 19 (FGF19, FGF15 in rodents), which circulates to the liver in portal blood, binds to the heterodimer FGF4-receptor/β-klotho, and triggers a signaling cascade to inhibit CYP7A1. When ileal reuptake of BAs is functionally impaired as in BA malabsorption syndrome or pharmacologically blocked with an IBAT inhibitor (right part of figure), BAs decrease and fecal BA excretion increases which may cause BA malabsorptive diarrhea. Decreased BA activation of ileal FXR results in reduced formation of FGF19 and diminished feed-back inhibition of BA synthesis. Increased BA synthesis via CYP7A1, reflected by increased serum C4, depletes the liver from cholesterol, which is compensated by increased cholesterol synthesis and the enhanced expression of low-density lipoprotein receptor (LDLR), which results in decreased serum LDL cholesterol.

Bile acids (BAs) are amphipathic molecules that are synthesized from cholesterol in the liver (**Figure 1**) (de Aguiar Vallim et al., 2013; Molinaro et al., 2018). Their primary function is to solubilize lipids into micelles, aiding digestion and absorption of fat. Once synthesized, they are conjugated with glycine or taurine and then excreted with bile into the small bowel from where about 95% are reabsorbed in the terminal ileum via the apical sodium-dependent bile salt transporter [ASBT, *SLC10A2*, also known as ileal BA transporter (IBAT)] (Dawson et al., 2003) and recirculated via the portal vein to the liver where they are taken up by the sodium-dependent taurocholate co-transporting peptide (NTCP, SLC10A1) (de Aguiar Vallim et al., 2013). This so-called enterohepatic circulation of BAs is shown in **Figure 2**. Of note, the related membrane proteins ASBT and NTCP preferentially transport conjugated BAs whereas unconjugated BAs may diffuse through the bowel mucosa and are taken up into the liver with the help of organic anion transporters.

The rate-limiting enzyme of BA synthesis, CYP7A1, is controlled by two different negative feedback pathways that are both regulated by binding of BAs to its nuclear receptor, farnesoid X receptor (FXR): (i) within the liver via the nuclear receptor small heterodimer partner (SHP) and (ii) from the ileum via fibroblast growth factor 19 (FGF19), which circulates to the liver in portal blood, binds to the heterodimer FGF4-receptor/β-klotho, and triggers a signaling cascade to inhibit CYP7A1 (**Figure 2**). When ileal reuptake of BAs is functionally impaired or pharmacologically blocked with an IBAT inhibitor, circulating BAs decrease and fecal BA excretion increases. Decreased BA activation of ileal FXR results in reduced formation of FGF19 and, in turn, increased BA synthesis, as reflected by increased levels of the serum BA precursor C4 (7α-hydroxy-4-cholesten-3-one). Enhanced conversion of cholesterol to BA results is compensated both by increased *de novo* biosynthesis and increased hepatic expression of low-density lipoprotein (LDL) receptor, which results in lowered circulating LDL cholesterol (**Figure 2**). The opposite biochemical changes, and decreased high-density lipoprotein (HDL) cholesterol probably due to decreased apolipoprotein (apo) A1 and increased scavenger receptor-B1 (SR-B1) expression, are observed by the administration of FXR activators such as obeticholic acid, which is a semisynthetic BA derivative (Nevens et al., 2016; Pencek et al., 2016). Of note, other semisynthetic BA derivatives were in rodents found to act on xenobiotic transporters such as multidrug-resistance protein (MRP) 3 (Al-Salami et al., 2008).

Because the ASBT inhibitors that are currently being tested have negligible systemic effects (i.e., they do not appear to affect ASBT expressed elsewhere, in particular in the biliary tree), we will use the term IBAT inhibitor in this review to refer to these ASBT inhibitors.

## Phase I Clinical Trials With IBAT Inhibitors

The mode of action of IBAT inhibitors has been demonstrated in three randomized double-blind placebo-controlled phase 1 trials. The first study used the IBAT inhibitor A4250 and included 40 and 24 healthy individuals, respectively, that were administered a single dose of A4250 (dose range: 0.1–10 mg) or A4250 for 1 week (1 or 3 mg once daily or 1.5 mg twice daily). A4250 decreased circulating FGF19 and increased C4 concentrations. Serum BA concentrations decreased consistently with increased fecal BA excretion (Graffner et al., 2016).

The second trial evaluated the IBAT inhibitor SHP626 (Volixibat) in 50 healthy subjects and in addition in 11 patients with type 2 diabetes mellitus /T2DM). SHP626 was administered in a dose range of 0.5–10 mg/day for 28 days. SHP626, as compared with placebo, increased mean total fecal BA excretion about ∼1.6–3.2 times in healthy volunteers and ∼8 times in patients with T2DM. With SHP626, mean C4 concentrations increased by ∼1.3–5.3-fold from baseline to day 28 in healthy volunteers and twofold in T2DM patients (Tiessen et al., 2018).

The third trial evaluated the IBAT inhibitor GSK2330672 in a 4-period crossover study in 16 Japanese subjects with single oral doses of GSK2330672 (10–180 mg) or placebo in each period. A dose-dependent tendency for total serum BAs to reduce and for serum C4 to increase was observed (Ino et al., 2018).

All three IBAT inhibitors showed similar adverse events: dose-dependent diarrhea (up to 50, 100, and 83% with A4250, SHP626, and GSK2330672, respectively) and abdominal pain (up to 33, 78, and 17% with A4250, SHP626, and GSK2330672, respectively) (Graffner et al., 2016; Ino et al., 2018; Tiessen et al., 2018).

## IBAT Inhibition for the Treatment of Constipation-Predominant Irritable Bowel Syndrome

Impaired or absent reuptake of BAs in the terminal ileum as seen in Crohn’s patients with active ileal disease or after terminal ileal resection may result in diarrhea. This clinical condition is nowadays termed type 1 BA malabsorption in contrast to idiopathic or type 2 BA malabsorption where impaired function of IBAT or impaired ileal feed-back regulation of BA synthesis may be the reason for BA diarrhea (Mottacki et al., 2016). A 2-week proof-of-concept study in patients with primary and secondary BA diarrhea indicated the potential benefit of enhancing FGF19 feedback signaling from the terminal ileum by administration of obeticholic acid (Walters et al., 2015). Idiopathic adult-onset BA malabsorption is not a rare finding and may often be the underlying cause of diarrhea-predominant irritable bowel syndrome (IBS-D) (Wedlake et al., 2009).

Conversely, pharmacological inhibition of IBAT might increase the number of bowel movements in patients with constipation-predominant IBD (IBD-C) or chronic idiopathic constipation (CIC). Indeed, this concept has successfully been tested using the IBAT inhibitor A3309 (Elobixibat) in European, United States and Japanese patients, respectively (**Supplementary Table 1**). In the first single-center randomized, double-blind, placebo-controlled study, 30 patients with CIC were randomized into five dose-levels (range: 0.1–10 mg/day) or placebo for 14 days (Simren et al., 2011). A3309 induced BA synthesis (C4) and reduced FGF19, as well as total and LDL cholesterol. Colonic transit time was reduced in the highest dose group and, overall, a trend for an increased number of bowel movements was observed (Simren et al., 2011). In a larger phase IIb randomized, double-blind, parallel-group, placebo-controlled trial, 190 patients with CIC were randomized to 5, 10, or 15 mg A3309 or placebo once daily (Chey et al., 2011). All patients randomized to A3309 noticed a significant improvement in spontaneous bowel movements (SBM) that was dose-dependent and maintained over 8 weeks. In addition, A3309 improved the serum lipid profile with significant decreases in total and LDL cholesterol in the 10- and 15-mg dose groups while HDL cholesterol or triglycerides did not change. The most common adverse events were abdominal pain and diarrhea, which occurred most commonly in the 15-mg A3309 group (Chey et al., 2011). A third randomized placebo-controlled study in 36 women with functional constipation administered 15 or 30 mg A3309 for 14 days. The IBAT inhibitor accelerated colonic transit and loosened stool consistency. Main adverse effects also in this trial were abdominal pain and diarrhea (Wong et al., 2011). In the fourth phase II trial with A3309, 163 Japanese patients were randomized to placebo or either 10 or 15 mg A3309 (Nakajima et al., 2018a). A dose of 10 mg/day was considered optimal; common adverse events included mild abdominal pain and diarrhea in the A3309 groups. Subgroup analysis indicated that A3309 was equally effective in patients with or without IBS-C (Nakajima et al., 2018a).

Recently, the combined results of two phase 3 trials with A3309 for CIC in Japanese patients have been published. The first was a randomized placebo-controlled 2-weeks trial with 15 mg/day of A3309 in 133 patients, the other an open label trial were 341 patients could titrate the dose of A3309 between 5 and 15 mg/day (Nakajima et al., 2018b). A3309 resolved constipation in the short-term trial and resulted in sustained improvement in bowel functions and quality of life in the 52-week treatment period where mild gastrointestinal disorders occurred in 40% of patients. A3309 also significantly reduced LDL cholesterol. Abdominal pain and diarrhea were reported in 19 and 13% in the 2-weeks, and in 24 and 15% in the 52 weeks trial, respectively (Nakajima et al., 2018b). Based on these studies, on January 20, 2018, A3309 was approved in Japan for the treatment of chronic constipation.

## Pre-Clinical Trials of IBAT Inhibitors in Mouse Models of Liver Diseases

The accumulation of BAs undoubtedly plays a major role in liver injury in cholestatic liver disease (Fickert and Wagner, 2017), and aberrant BA profiles have been found in various studies of human obesity and metabolic syndrome-related diseases such as type 2 diabetes mellitus (T2DM) and NAFLD/NASH (Molinaro et al., 2018). Importantly, BAs both by intestinal and hepatic FXR activation in addition to cholesterol turnover via apoA1, LDLR, and SR-B1 also regulate triglyceride metabolism by pathways that involve FXR, SHP and sterol responsive element binding protein 1c (SREBP-1c) and FG15/FGF19 (reviewed in Chavez-Talavera et al., 2017). Thus, both by depleting the BA pool and interruption of intestinal FXR activation, IBAT inhibitors may improve liver function both in cholestatic and/or non-alcoholic fatty liver disease (NAFLD) and non-alcoholic steatohepatitis (NASH) (Arab et al., 2017). This hypothesis is supported by pre-clinical studies using animal models of cholestatic and fatty liver disease. IBAT inhibitors SC-435 and A4250 were investigated in *Mdr2^-/-^* mice, which is an established model of sclerosing cholangitis (Fickert et al., 2004), and SC-435 was also studied in high-fat diet (HFD)-induced fatty liver disease. SC-435 was previously described to lower serum cholesterol and reduce atherosclerosis in dogs, apolipoprotein E (apoE) deficient mice (Bhat et al., 2003), and guinea pigs (West et al., 2003).

In one study in *Mdr2^-/-^* mice, 30-day-old female mice were fed HFD containing 0.006% SC-435 for 14 days. Compared with untreated mice, SC-435 resulted in an eightfold increase in fecal BA excretion, with a concomitant 90-fold reduction of serum BA concentrations and reduction of serum alanine transferase (ALT), total bilirubin, and serum alkaline phosphatase (ALP) levels, indicating anti-inflammatory and anti-cholestatic effects. Liver histology improved and the extent of fibrosis decreased concomitant with reduced expression of hepatic profibrotic genes (Miethke et al., 2016). In another study, 8-week-old *Mdr2^-/-^* (*Abcb4^-/-^*) mice received either a diet supplemented with A4250 (0.01% w/w) or a chow diet. A4250 significantly reduced serum ALT, ALP and BA levels, hepatic expression of pro-inflammatory (*Tnf-α, Vcam1, Mcp-1*) and pro-fibrogenic (*Col1a1, Col1a2*) genes, cholestatic injury in liver histology and bile duct proliferation as shown by immunohistochemistry for cytokeratin 19 (CK19). Furthermore, A4250 significantly reduced bile flow and biliary BA output while biliary ${\mathrm{HCO}}_{3}^{–}$ and phospholipid secretion was preserved. Importantly, A4250 profoundly increased fecal BA excretion without causing diarrhea (Baghdasaryan et al., 2016). Both these studies thus showed that IBAT inhibition improved biochemical and histological features of sclerosing cholangitis, indicating that this drug treatment could be potentially beneficial for human cholestatic liver disease.

SC-435 then was studied in male C57Bl/6J mice fed HFD for 16 weeks. To show its protective effect against NAFLD, SC-435 was administered during the whole 16 weeks HFD period while administration of SC-435 was first started after 12 weeks to show its ability to reverse the effects of HFD. SC-435 induced mRNA expression of BA synthesis genes in the liver and reduced mRNA expression of ileal BA-responsive genes, including FGF15 (murine homolog of human FGF19). SC-435 also restored glucose tolerance, reduced hepatic triglyceride and total cholesterol concentrations, and improved NAFLD activity score in HFD-fed mice. These changes were associated with reduced hepatic expression of lipid synthesis genes and normalized expression of the central lipogenic transcription factor, *Srebp1c* (Rao et al., 2016). Despite fourfold increased fecal BA excretion during SC-435 treatment, total liver BAs did not change. Rather, hepatic BA profiles in HFD fed mice shifted from a more hydrophilic and FXR antagonistic/non-agonistic composition with 42% 6-hydroxylated (murine) BAs to are more hydrophobic and FXR agonistic composition (17% 6-hydroxylated BAs). Of note, the phenotype of HFD-fed *Asbt^-/-^* mice was very similar to HFD and SC-435 treated wild-type mice, further supporting the concept that interruption of the enterohepatic circulation of BAs might protect against NAFLD (Rao et al., 2016).

## Phase 2 Clinical Trials With IBAT Inhibitors for the Treatment of Cholestatic Pruritus

Cholestatic liver diseases arise from impaired hepatobiliary production and excretion of bile, which cause bile constituents to enter the circulation. Injuries to bile ducts or hepatocytes can lead to a range of clinical presentations, from isolated abnormalities in liver biochemistry, to liver failure or hepatobiliary malignancy; congenital, immunologic, structural (obstructive/vascular), and toxic factors can all contribute to disease. In response to injury, mature cholangiocytes and hepatocytes proliferate, which may lead to periductular fibrosis, biliary fibrosis, and cirrhosis. Disease progression and the efficacy of repair depend on etiology and the individual’s response to injury. Most common cholestatic liver diseases in adults are primary biliary cholangitis (PBC), primary sclerosing cholangitis (PSC) and drug-induced cholestasis and in children, Alagille syndrome, biliary atresia, and progressive familial intrahepatic cholestasis (PFIC) (Hirschfield et al., 2010).

Pruritus (itch) is a frequent and troublesome symptom in patients with cholestatic liver disease, in particular in PBC where it is seen in 60–70% of patients at some point during the disease process (Talwalkar et al., 2003). The pathogenesis of cholestatic pruritus is complex and several putative pruritogens have been proposed, including circulating BAs (Beuers et al., 2014). Recently, autotaxin was identified as a major contributor to cholestatic pruritus (Kremer et al., 2010, 2012). Limiting the amount of circulating BAs by the administration of BA binding resins (BA sequestrants) is an established treatment modality of cholestatic pruritus and despite modest evidence of its efficacy and poor tolerability profile, cholestyramine is the only drug licensed for the treatment of PBC-related pruritus (European Association for the Study of the Liver, 2017). Rifampicin as second-line therapy for cholestatic pruritus has a success rate of about 50% in clinical practice but is hampered by hepatotoxic side effects (Prince et al., 2002). Other drug therapies including opiate antagonists (as third-line therapy), selective serotonin uptake inhibitors and gabapentin are less well-documented (European Association for the Study of the Liver, 2009). Nasobiliary drainage effectively reliefs from cholestatic pruritus but is invasive and uncomfortable (Hegade et al., 2016).

Although the role of BAs in the pathogenesis of cholestatic pruritus remains unclear (as BA levels in serum, urine, or skin do not correlate with severity of pruritus) (Beuers et al., 2014), depleting the BA pool by the administration of IBAT inhibitors might improve itching. This concept has been tested in pilot and phase II trials in both PBC and pediatric cholestatic liver diseases (**Supplementary Table 1**).

The IBAT inhibitor GSK2330672 was tested in a double-blind, randomized, placebo-controlled, crossover trial in two United Kingdom medical centers (Hegade et al., 2017). In this study, 22 PBC patients with refractory, severe pruritus received 14 days each of placebo or GSK2330672 (45–90 mg twice daily) with randomization to the sequence of placebo and GSK2330672. Pruritus as estimated by visual analog scale (VAS), PBC-40 itch domain and 5-D itch scale improved by placebo but the IBAT inhibitor was significantly more efficient in any of these parameters and the effect was independent of the course of treatment. GSK2330672 reduced serum BAs, FGF19 and autotaxin and increased C4 concentrations. Of note, 33% of the patients receiving GSK2330672 reported diarrhea and a substantial number of patients reported abdominal pain, but no study subjects withdraw from treatment (Hegade et al., 2017). The phase 2 trial of GSK2330672 administration for the treatment of pruritus in patients with primary biliary cholangitis (GLIMMER) trial is currently recruiting 118 patients for a 16-week treatment (NCT02966834).

The IBAT inhibitor SHP625 (LUM001, Lopixibat, Maralixibat) was administered for 12 weeks in 66 PBC patients at a dose of 10–20 mg once daily (Mayo et al., 2016). This compound was not more efficient on pruritus than placebo, which markedly decreased all itching scores. In patients randomized to SHP625, serum BA reductions and C4 increases reached nominal significance; however, a higher incidence of diarrhea and abdominal pain was reported, resulting in withdrawal in 2/42 patients (Mayo et al., 2016). SHP625 has also been tested in 27 patients with PSC but no data are available as yet.

In a recent pilot study, we assessed the tolerability and effect on pruritus of A4250 in patients with PBC. Nine patients with ongoing long-term BA sequestrant treatment of cholestatic pruritus were treated with A4250 for 4 weeks after a 2-week wash out of the resins. Patients’ pruritus was also here assessed by VAS, 5-D itch scale and the PBC40 pruritus module. All nine patients exposed to A4250 (0.75 mg, *n* = 4; 1.5 mg, *n* = 5) reported a rapid (within 48 h) and substantial improvement in pruritus that in particular restored normal sleep at night in the four patients who finished the whole 4-week treatment period with A4250. Also scratch marks on the skin diminished in the patients who had this sign of severe pruritus. However, five of the nine patients exposed to A4250 withdrew already during the first week due to diarrhea and abdominal pain, which indicates that the starting dose of A4250 might have been too high (Al-Dury et al., 2018).

The effect of IBAT inhibitors SHP625 and A4250 on pruritus was also investigated in various cholestatic pediatric liver diseases. Thirty-seven patients with Alagille syndrome were randomly assigned to receive either SHP625 (70, 140, or 280 μg/kg/day once-daily) or placebo for 13 weeks. Pruritus was assessed using a novel pediatric version of the Itch Report Outcome (ItchRO^TM^) and a clinician-reported scratch scale, both of which grade the severity of reported itching on a scale of 0–4 (none to severe). The percentage of patients with a decrease in the degree of itching from baseline to week 13 was higher among those who received SHP625 than those who received placebo for both scales (ItchRO: 65 vs. 25%; clinician-reported scratch scale: 76 vs. 25%, respectively). Adverse events were similar between SHP625 and placebo recipients (Shneider et al., 2017).

In another study with SHP625, this IBAT inhibitor was administered for 48 weeks to 33 children with PFIC (age: 1–13 years); the SHP625 dose was escalated from 14 to 280 μg/kg/day over 13 weeks (depending on tolerability) and maintained for up to 50 weeks. Efficacy data were available in 26/29 participants who reached week 48 (PFIC1, *n* = 6; PFIC2, *n* = 20), of whom 23 were receiving maralixibat 280 μg/kg/day. In 6/20 participants with PFIC2 (30%), efficacy profiles over 48 weeks indicated clinically significant response to SHP625: BA levels normalized to ≤8.5 μmol/L (*n* = 4) or reduced by ≥70% (*n* = 2); ItchRO scores showed no pruritus (*n* = 2) or improved by ≥1.0 points (*n* = 4). Treatment-emergent adverse events were reported in all 33 participants, were judged related to SHP625 in 22, serious in 15 and led to discontinuation in 1. The most frequently reported treatment related adverse events were pyrexia (*n* = 15), diarrhea (*n* = 14), cough (*n* = 13), abdominal pain (*n* = 10), and vomiting (*n* = 10) (Thompson et al., 2017).

A4250 was administered to 20 pediatric patients (12 males, 8 females, age: 1–17 years) with PFIC type 1, 2, or 3 (*n* = 13: 3 re-entries), Alagille syndrome (*n* = 6), biliary atresia (*n* = 3), and intrahepatic cholestasis (*n* = 2; 1 re-entry). A4250 was administered orally once daily for 4 weeks at five doses (10–200 μg/kg). Serum BAs decreased significantly (*p* < 0.008). Patients reported an improvement in pruritus and sleep, and reductions in VAS-Itch were significantly correlated with reductions in serum BAs. No serious adverse events were deemed treatment related, and most adverse events, including some increased transaminases, were transient (Sturm et al., 2017).

## Phase 2 Clinical Trials With IBAT Inhibitors for the Treatment of Non-Alcoholic Steatohepatitis (NASH)

In the T2DM cohort of the phase 1 study with SHP626, trends were observed toward increased levels of HDL cholesterol, decreased levels of serum triglycerides and reductions in fasting glucose levels that were considered suggestive of improvements in both lipid and glucose homeostasis (Tiessen et al., 2018). T2DM and obesity are the most common underlying disorders in NAFLD and its serious form NASH. Recently, SHP626 has gained fast track designation by the food and drug administration (FDA) and is now being tested in a phase II double-blind, randomized, placebo-controlled, dose-finding trial with 292 patients. SHP626 or placebo is being orally administered for 48 weeks at a daily dose of 5, 10, or 20 mg. The primary outcome of this study (NCT02787304) is a reduction from baseline of the NAFLD activity score of at least two points, without worsening of fibrosis, at 48 weeks. Other outcome parameters are changes in liver fat content (as measured by magnetic resonance imaging (MRI), fibrosis state, liver enzymes, bilirubin and metabolic biochemistry.

In summary, so far IBAT inhibition has only emerged as treatment option in chronic constipation, gaining its benefit by inducing BA malabsorptive diarrhea. For cholestatic pruritus, published data, while somewhat inconclusive, support the hypothesis that the interruption of the enterohepatic circulation of BAs via IBAT inhibition has the potential to relieve patients from itch. However, the substantial incidence of abdominal pain and diarrhea may limit its applicability, at least in adults and during long-term treatment. Careful drug administration regiments might help to improve tolerability, in particular very slowly increasing doses. Certainly, efficacy and tolerability need to be compared to BA sequestrants although it seems to be very difficult to do this under placebo-controlled conditions.

## Perspective

Depletion of the BA pool from potentially toxic compounds by interrupting their enterohepatic circulating with drugs that have very low systemic enrichment is conceptionally interesting and it might turn out that patients with liver inflammation as in NASH might have the highest clinical benefit. Also, these compounds might have a role in combination therapies, e.g., with nor-ursodeoxycholic acid (Fickert et al., 2017), where reabsorption of conjugated in contrast to *de novo* absorption of unconjugated BA is less desirable as this would result in higher enrichment of the unconjugated compounds in the BA pool.

## Author Contributions

SA-D and H-UM wrote this review together.

## Conflict of Interest Statement

H-UM had consulted for and received material support from Albireo and Intercept. The remaining author declares that the research was conducted in the absence of any commercial or financial relationships that could be construed as a potential conflict of interest. The handling Editor and reviewer HA-S declared their involvement as co-editors in the Research Topic, and confirm the absence of any other collaboration.
